# Novel bulking agent for faecal incontinence

**DOI:** 10.1002/bjs.7699

**Published:** 2011-09-16

**Authors:** C Ratto, A Parello, L Donisi, F Litta, V De Simone, L Spazzafumo, P Giordano

**Affiliations:** 1 Department of Surgical Sciences, Catholic UniversityRome, Italy; 2 Centre of Statistics, Istituto Nazionale di Ricovero e Cura per AnzianiAncona, Italy; 3 Department of Surgery, Whipps Cross University HospitalLondon, UK

## Abstract

**Background:**

Various injectable bulking agents have been used for the treatment of faecal incontinence (FI). However, encouraging early results are not maintained over time. This study aimed to assess short- and medium-term results of a new bulking agent for the treatment of FI.

**Methods:**

The Gatekeeper™ prosthesis comprises a thin solid polyacrylonitrile cylinder that becomes thicker, shorter and softer within 24 h after implantation. Fourteen patients with FI underwent treatment with Gatekeeper™ under local anaesthesia. Four prostheses were implanted in the intersphincteric space in each patient, under endoanal ultrasound guidance. Number of episodes of major FI, Cleveland Clinic FI score (CCFIS), Vaizey score, anorectal manometry, endoanal ultrasonography (EUS), health status and quality of life (Short Form 36 and Faecal Incontinence Quality of Life questionnaires) were assessed before and after treatment.

**Results:**

Mean(s.d.) follow-up was 33·5(12·4) months. There were no complications. There was a significant decrease in major FI episodes from 7·1(7·4) per week at baseline to 1·4(4·0), 1·0(3·2) and 0·4(0·6) per week respectively at 1-month, 3-month and last follow-up (*P* = 0·002). CCFIS improved significantly from 12·7(3·3) to 4·1(3·0), 3·9(2·6) and 5·1(3·0) respectively (*P* < 0·001), and Vaizey score from 15·4(3·3) to 7·1(3·9), 4·7(3·0) and 6·9(5·0) respectively (*P* = 0·010). Soiling and ability to postpone defaecation improved significantly, and patients reported significant improvement in health status and quality of life. At follow-up, manometric parameters had not changed and EUS did not demonstrate any prosthesis dislocation.

**Conclusion:**

The Gatekeeper™ anal implant seemed safe, reliable and effective. Initial clinical improvement was maintained over time, and follow-up data were encouraging. Copyright © 2011 British Journal of Surgery Society Ltd. Published by John Wiley & Sons, Ltd.

## Introduction

Faecal incontinence (FI) is a common problem that can present with a wide variety of symptoms ranging from involuntary but recognized passage of gas, liquid or solid stool to unrecognized anal leakage of mucus, fluid or stool. Depending on the degree of symptoms, FI can be a highly distressing and socially incapacitating problem. The wide variety of aetiologies and difficulty in accurately defining the cause of the problem make treatment difficult. Appropriate treatment relies on accurate diagnosis and careful patient selection. Various injectable anal bulking agents have been used to treat FI^1^–^7^, but procedures have not been standardized, and the most effective site of placement as well as the amount of agent to be delivered has yet to be established. Moreover, depending on the material used, dislocation, migration and absorption can occur. Owing to significant differences between bulking agents and clinical conditions in which they are used, results are controversial and difficult to interpret. It seems that early positive results of anal bulking agents are often not confirmed in the long term.

This aim of this study was assess the short- and medium-term results of a new bulking agent used for the treatment of FI.

## Methods

This study, carried out at the Department of Surgical Sciences, Catholic University, Rome, was approved by the institutional ethics committee.

### Anal bulking agent

**Fig. 1:**
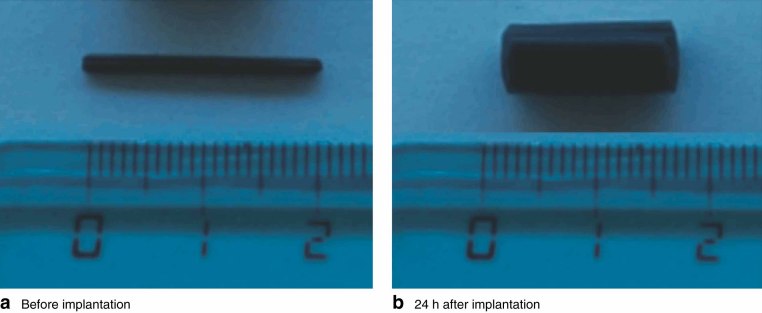
Gatekeeper™ prosthesis **a** before implantation and **b** 24 h after implantation

Gatekeeper™ prostheses (originally from Medtronic, Minneapolis, Minnesota, USA; now from THD, Correggio, Italy) were used as anal bulking agent. These are thin solid cylinders (length 21 mm, diameter 1·2 mm) of HYEXPAN™ (polyacrylonitrile), a hydrophilic material that, within 24 h of implantation in contact with human tissue, changes shape and volume, becoming thicker (diameter 7 mm), shorter (length 17 mm) and of softer consistency (*Fig. 1*). Their final shape yields a 720 per cent volume increase compared with the volume inserted. The material is identifiable on palpation and ultrasonography.

### Selection criteria and preoperative data collection

Patients with at least a 6-month history of episodes of FI (soiling or incontinence to liquid and/or solid stools) occurring at least once a week that had failed to improve with conservative measures were invited to participate. Patients with isolated incontinence to gas, risk of significant postoperative complications, including uncontrolled diabetes, anal sepsis, inflammatory bowel diseases with anorectal involvement or any colorectal cancer undergoing active treatment, were excluded. Patients with an isolated external anal sphincter (EAS) defect demonstrated on endoanal ultrasonography (EUS) were also excluded. Patient selection was based on data collected from the history, physical examination, continence diary recorded for 14 days (assessing incontinence episodes to gas, liquid and solid stool; postevacuation soiling episodes; and inability to postpone defaecation), Cleveland Clinic FI score (CCFIS)^8^ and Vaizey score^9^. Further information on health status and quality of life was obtained from questionnaires: Short Form 36 health survey (SF-36®; QualityMetric, Lincoln, Rhode Island, USA)^10^ and Faecal Incontinence Quality of Life (FIQL)^11^. All patients were assessed with anorectal manometry and EUS.

### Implantation technique

**Fig. 2:**
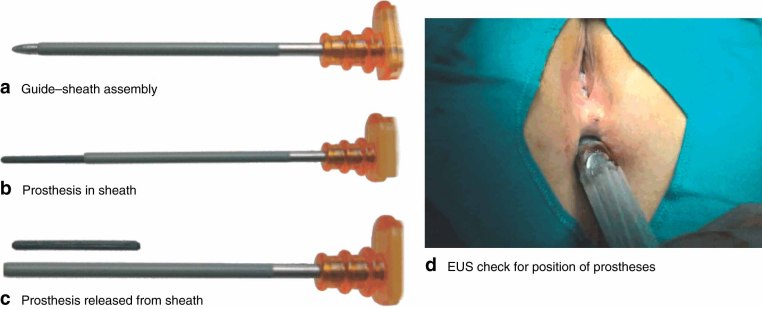
Implantation of Gatekeeper™ prosthesis: **a** metal guide and external sheath assembled together; **b** following removal of the metal guide, the prosthesis is introduced through the sheath; **c** the prosthesis is released from the sheath; **d** the position of the prosthesis is checked by endoanal ultrasonography (EUS) at the end of the procedure

The procedure was performed as a day case, under local anaesthesia using a posterior perineal block with the patient placed in the lithotomy position. Four 2-mm skin incisions were made at 3, 6, 9 and 12 o'clock positions in the perianal area 2 cm from the anal verge. With an Eisenhammer retractor inserted in the anal canal, a dedicated introducer formed by an introducer guide and an external sheath (*Fig. 2*) was tunnelled from the skin incision to the intersphincteric margin, and advanced into the intersphincteric space until the tip of the introducer reached the level of the puborectalis muscle. The introducer guide was removed, leaving the sheath in the intersphincteric space. The prosthesis was inserted into the lumen of the introducer sheath and advanced. When the prosthesis reached the middle–upper anal canal the introducer sheath was removed, leaving the prosthesis in place. The same procedure was repeated for all four positions. All prosthesis placement steps were carried out under direct vision and under EUS guidance (Model 1850® equipped with a system for three-dimensional reconstruction; B-K Medical, Herlev, Copenhagen, Denmark). At the end of procedure, the correct positioning of the prostheses was confirmed by EUS (*Fig. 2*). After the procedure patients were discharged home, with advice to avoid heavy physical activity for at least 48 h. All patients received oral antibiotic prophylaxis for 3 days.

### Follow-up

Patients were reviewed in outpatients at 7, 30 and 90 days, and 6 months thereafter. All patients were recalled for further evaluation at the time of study closure. Anorectal manometry was performed at 30 and 90 days, and at final follow-up. Each patient kept a continence diary for 14 days before the follow-up appointments 30 and 90 days after operation and the last follow-up. Data obtained from the continence diary were used to calculate the CCFIS and Vaizey score. At the last follow-up appointment patients were also asked to complete the SF-36® and FIQL questionnaires. All adverse events occurring during the first 3 months after the procedure were recorded.

### Study endpoints

The primary endpoint was safety of the surgical technique assessed as intraoperative and postoperative complications, prosthesis displacement and any other morbidity. Secondary endpoints were therapeutic efficacy of Gatekeeper™ injection in terms of improvement in FI symptoms, changes in manometric parameters, and changes in health status and quality of life.

### Statistical analysis

Continuous data were presented as mean(s.d.), and compared using the Wilcoxon test. Fisher's exact test was used for analysis of categorical data. *P* < 0·050 was considered statistically significant. Analyses were carried out with SPSS® version 12.0 software for Windows® (SPSS, Chicago, Illinois, USA).

## Results

Fourteen consecutive patients (6 men, 8 women) with a mean age of 63·5(17·0) (range 28–83) years were enrolled from May 2005 to January 2008. *Table 1* shows the history, and baseline clinical and quality-of-life data. All eight women enrolled in the study had given birth, two by caesarean and six by vaginal delivery; four of the vaginal deliveries included an episiotomy.

**Table 1:** Baseline clinical features

	No. of patients*
History	
Anal surgery‡	5
Anal trauma	2
Abdominal surgery	3
Diabetes	3
Pelvic radiotherapy	1
Faecal incontinence	
Age at onset (years)†	53·9(18·6)
Duration (months)†	11·6(8·6)
Ability to defer defaecation (min)†	6·1(4·9)
< 5	0
5–10	2
11–20	4
> 20	8
Postdefaecation soiling	
Often/always	9
Sometimes	2
Never/rarely	3
Incontinence to gas (episodes/week)†	21·2(15·1)
Incontinence to liquid (episodes/week)†	6·2(6·0)
Incontinence to solids (episodes/week)†	0·9(1·4)
Need to wear pad	
Always/often	6
Sometimes	0
Rarely/never	8
Lifestyle alteration	
Always/often	12
Sometimes	1
Rarely/never	1
CCFIS†	12·7(3·3)
Vaizey score†	15·4(3·3)
Quality of life†	
SF-36®	
Physical function	62·8(22·3)
Role physical	25·0(41·6)
Bodily pain	84·3(24·9)
General health	32·0(24·4)
Vitality	50·7(22·9)
Social function	29·5(23·3)
Role emotional	19·0(36·3)
Mental health	44·0(26·5)
FIQL score	
Lifestyle	1·76(0·47)
Coping and behaviour	1·37(0·38)
Depression and self-perception	2·07(0·57)
Embarrassment	1·53(0·64)

* Unless indicated otherwise;

† values are mean(s.d.).

‡ Lateral internal sphincterotomy in three patients, haemorrhoidectomy in three, fistulotomy in two. CCFIS, Cleveland Clinic faecal incontinence score; FIQL, Faecal Incontinence Quality of Life.

Postevacuation soiling was reported to occur always in nine patients, sometimes in two, rarely in two and never in one patient. The mean number of major incontinence episodes was 7·1(7·4) per week overall, 6·2(6·0) per week to liquids and 0·9(1·4) per week to solids. The mean CCFIS was 12·7(3·3) (range 7–18); only three patients had a CCFIS below 10. The mean Vaizey score was 15·4(3·3) (range 11–21). Patients could postpone defaecation for a mean of 6·1(4·9) min. EUS demonstrated no sphincter injury in eight patients, an isolated internal anal sphincter (IAS) defect in four, and a combined IAS and EAS defect in two patients (the latter patients both had an episiotomy during childbirth). Baseline anal manometric data are reported in *Table 2*.

**Table 2:** Anorectal manometry data

		Follow-up			
			
	Baseline	1 month	3 months	Last	P*
Functional anal canal length (cm)	3·0(1·2)	3·6(1·0)	3·5(1·1)	3·5(0·8)	0·065
Maximum resting pressure (mmHg)	79·0(30·8)	81·7(31·2)	81·0(29·2)	73·8(33·8)	0·872
Mean resting pressure (mmHg)	36·1(14·0)	37·7(15·0)	38·6(17·6)	34·5(16·5)	0·910
Squeeze pressure (mmHg)	90·5(66·4)	103·5(63·6)	93·0(58·9)	76·8(59·8)	0·102
Threshold rectal sensation (ml)	59·3(31·7)	73·4(44·1)	73·9(42·0)	75·0(38·0)	0·115
Urge rectal sensation (ml)	105·6(42·8)	121·1(55·3)	124·6(45·4)	131·1(49·9)	0·097
Rectal maximum tolerated volume (ml)	153·9(52·5)	181·4(74·5)	179·3(68·7)	190·0(60·5)	0·084

Values are mean(s.d.).

* Baseline *versus* 1 month *versus* 3 months *versus* last follow-up (Wilcoxon test). There were no significant differences between follow-up and baseline values.

All procedures were carried out successfully as a day case. Mean duration of operation was 35(7) min, including local anaesthesia, surgical procedure and control EUS. In all patients, the procedure proved to be easy and safe, and EUS confirmed accurate prosthesis placement. There were no intraoperative complications.

### Postoperative features

There was no postoperative morbidity. None of the patients experienced any degree of local or systemic sepsis, fever or pain. There was no evidence of any acute or chronic inflammatory response around the implanted prostheses. Neither prosthesis dislodgement nor mucosal/skin alteration (fistula, ulceration) was noted. Patients experienced no anal discomfort either at rest or during defaecation.

### Follow-up

Overall median follow-up was 33·5(12·4) (range 5–48) months. One week after Gatekeeper™ implantation, clinical assessment by digital examination and EUS showed the absence of acute inflammation at the prosthesis sites. All but one patient reported a significant improvement and regarded the treatment as successful. In one patient with an IAS defect secondary to lateral internal sphincterotomy the number of episodes of FI remained relevant (28 per week at baseline, 14 per week at 1 month and 12 per week at 3 months); 5 months after Gatekeeper™ implantation this patient underwent successful sacral nerve stimulation and was excluded from further analysis.

**Fig. 3:**
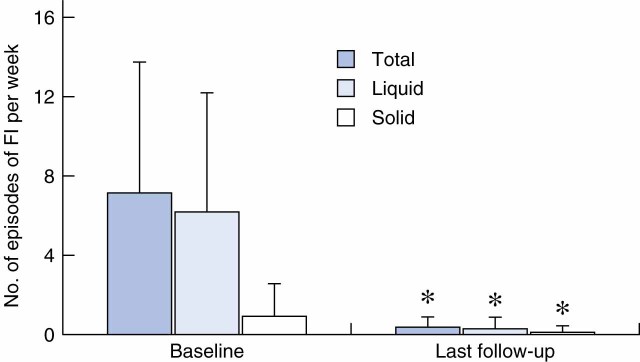
Mean(s.d.) number of episodes of major faecal incontinence (FI) overall, to liquid and to solid faeces at baseline and last follow-up after Gatekeeper™ implantation. *P < 0·050 *versus* baseline (Wilcoxon test)

**Fig. 4:**
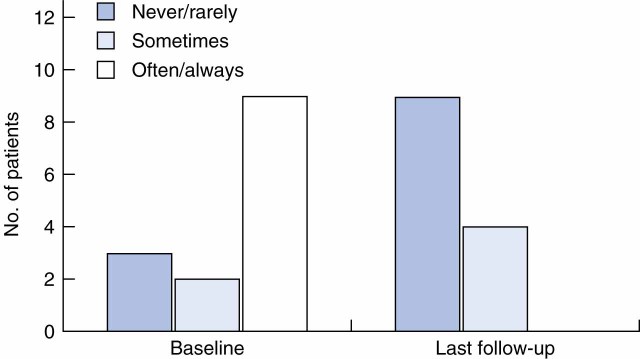
Number of patients affected by soiling and symptom frequency at baseline and last follow-up after Gatekeeper™ implantation. *P* = 0·028 (Fisher's exact test)

The mean total number of episodes of major FI decreased significantly immediately after surgery and the improvement was maintained over time, with a change from 7·1(7·4) per week before operation to 1·4(4·0), 1·0(3·2) and 0·4(0·6) per week at 1-month, 3-month and last follow-up respectively (*P* = 0·002). Findings were similar when episodes of incontinence to liquids or solids were analysed separately (*Fig. 3*). Postevacuation soiling and ability to postpone defaecation also improved significantly: before treatment three of 14 patients never or rarely experienced postevacuation soiling, whereas nine of 13 patients reported absence of postevacuation soiling at the last follow-up (*P* = 0·028) (*Fig. 4*). Defaecation could be delayed for a significantly longer period following treatment, from a baseline of 6·1(4·9) min to 21·9(13·8) min at last follow-up (*P* < 0·031).

**Fig. 5:**
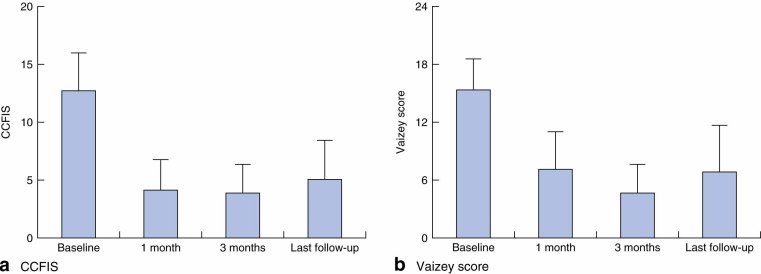
Mean(s.d.) **a** Cleveland Clinic faecal incontinence score (CCFIS) and **b** Vaizey score at baseline and during follow-up after Gatekeeper™ implantation. **a** *P* < 0·001, **b** *P* = 0·010 (Wilcoxon test)

Mean CCFIS was significantly reduced at the 1- and 3-month and last follow-up (*P* < 0·001) (*Fig. 5a*); only two of 13 patients had a CCFIS higher than 7 at the final evaluation. Mean Vaizey score changed from a baseline of 15·4(3·3) to 7·1(3·9), 4·7(3·0) and 6·9(5·0) at 1-month, 3-month and last follow-up respectively (*P* = 0·010) (*Fig. 5b*).

There was no correlation between anal sphincter defect on EUS and clinical outcome, with no differences between the subset of patients without sphincter lesions and those with sphincter disruption.

Mean anal manometric values did not change compared with baseline during follow-up (*Table 2*). A slight increase was noted in mean functional anal canal length and rectal sensation, but there were no statistically significant changes.

**Fig. 6:**
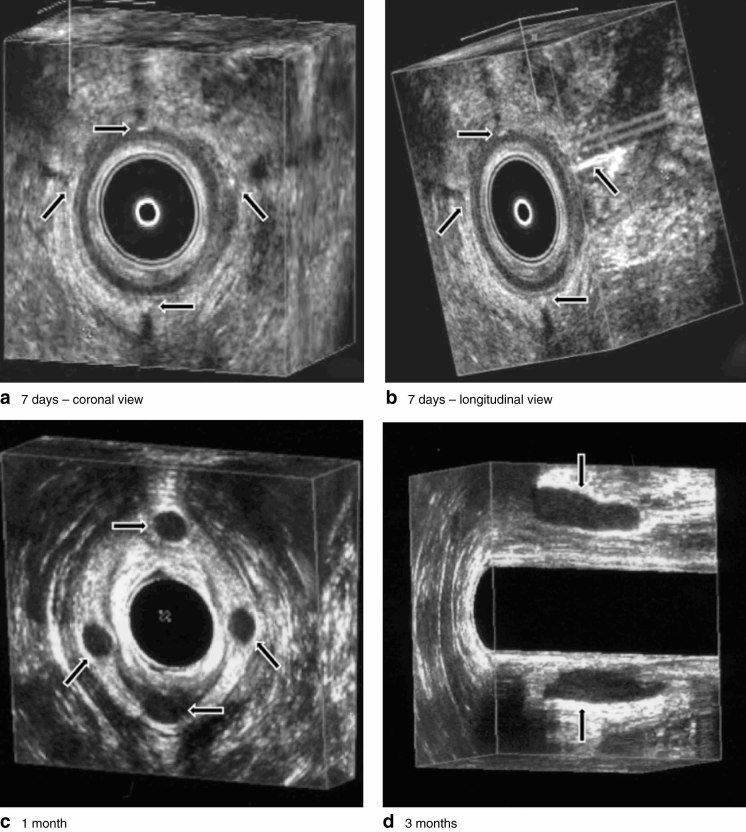
Endoanal ultrasound imaging at **a,b** 7 days (**a** coronal view, **b** longitudinal view), **c** 1 month and **d** 3 months after implantation of Gatekeeper™ prostheses (arrows)

One week after implantation, EUS confirmed the correct position of the prostheses. EUS also demonstrated the shape modification of prostheses following implantation; they appeared thicker, slightly shorter and anechoic. During follow-up, no inflammatory reactions were documented by EUS around the implanted prostheses, and there was no evidence of prosthesis displacement (*Fig. 6*).

**Fig. 7:**
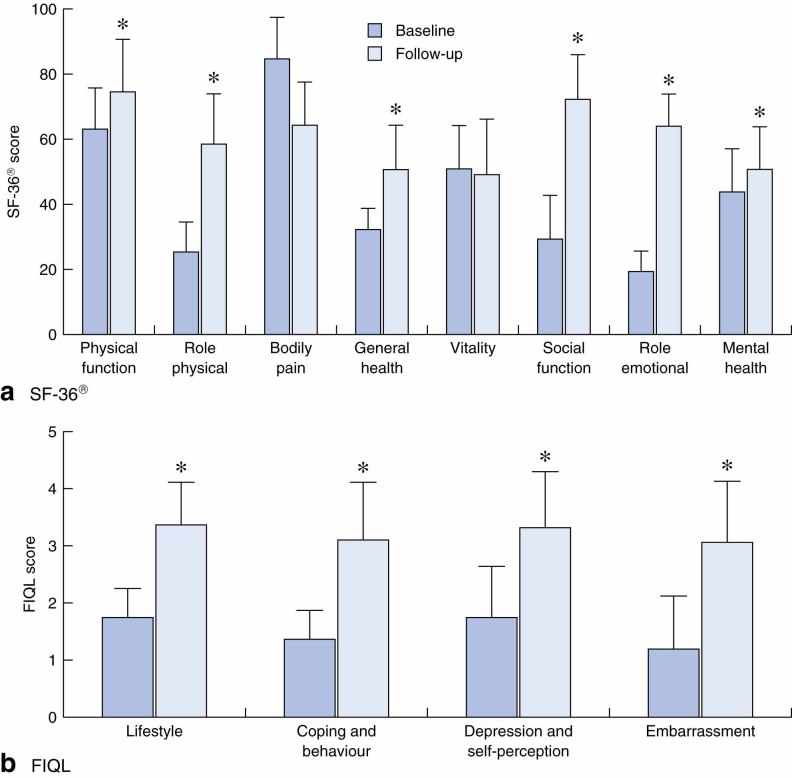
Mean(s.d.) scores on **a** Short Form 36 (SF-36®) health survey and **b** Faecal Incontinence Quality of Life (FIQL) questionnaire at baseline and last follow-up after Gatekeeper™ implantation. *P < 0·050 *versus* baseline (Wilcoxon test)

After implantation of Gatekeeper™, there was a significant increase in mean scores in the physical function, role physical, general health, social function, role emotional and mental health domains of the SF-36® at the last follow-up (*P* = 0·002, *P* = 0·001, *P* = 0·010, *P* < 0·001, *P* < 0·001 and *P* = 0·001 respectively) (*Fig. 7a*). All FIQL questionnaire items showed a significant improvement in values at final follow-up compared with baseline: lifestyle (*P* = 0·001), coping and behaviour (*P* < 0·001), depression and self-perception (*P* < 0·001) and embarrassment (*P* = 0·001) (*Fig. 7b*).

## Discussion

At the time this study was designed, Gatekeeper™ was used only in patients with gastro-oesophageal reflux disease (GORD). In 2008, however, the commercial licence for GORD was discontinued and enrolment for the present study was interrupted. The patients already enrolled were followed up according to the study protocol. In the meantime another company acquired the production line and recently obtained the EC mark of approval specifically for the use of Gatekeeper™ in FI, making this new bulking agent available on the market again.

Gatekeeper™ prostheses are made of a unique material (HYEXPAN™) that is solid at the time of delivery, but slowly absorbs water and expands once implanted. Within 48 h the prosthesis has reached its final size and shape. At this stage the consistency of the material has changed from hard to soft, giving the implant a pliable texture that makes it compliant to external pressures without losing its original shape. For these reasons it was decided to place the implants in the intersphincteric space, in the belief that this would achieve a more effective distribution of the bulking effect than would be achieved with submucosal positioning, and thus exploiting the physical characteristic of the implant most effectively. The intersphincteric location should also minimize the potential risk of erosion, ulceration, fistulation of the anal canal and possible displacement of the prosthesis. This is particularly important in view of the solid state of the prostheses at the time of implantation. In this series there were no complications related to implantation of the prostheses, and ultrasonographic surveillance for a mean of almost 3 years confirmed that none of the implants had become displaced. The ultrasound results also showed that the size of all prostheses remained virtually unchanged over time, thus confirming the durability of the Gatekeeper™. The present cohort included not only patients with an intact IAS but also those with an IAS tear, or both IAS and EAS defects; patients with isolated EAS defects, however, were excluded. The prostheses were placed in the same position in all patients, irrespective of the location of the sphincter lesion.

Skin incisions were made about 2 cm away from the anal verge to minimize the risk of wound contamination during bowel movements. The non-linear tunnelling through the soft subcutaneous tissues to reach the intersphincteric plane from the skin incision should also avoid possible prosthesis extrusion along the track. Prosthesis placement was performed under EUS guidance, to control the procedure step by step and ensure correct positioning of the prostheses. The operator could easily reach the intersphincteric space and decide on the exact position for each prosthesis. Moreover, the introducer could be followed by direct vision and digital palpation, and visualized by EUS. Therefore, lesions in the rectoanal mucosal/submucosal layer could be avoided. The contribution of EUS during Gatekeeper™ placement was fundamental to guiding placement of the prosthesis. However, the authors do not believe that it is necessarily mandatory; the procedure could be performed safely under digital guidance by an experienced clinician. Four prostheses were always implanted; this choice was arbitrary but seemed effective. The prostheses were placed at 3, 6, 9 and 12 o'clock positions for convenience but it is likely that, provided the implants are inserted correctly and distributed equally around the anal canal, the actual position may not influence the outcome. Whether the number of prostheses implanted influences the outcome is not clear at this stage.

In recent literature reviews, Vaizey and Kamm^12^ and Vaizey and Maeda^13^ analysed available data on the various bulking agents used in FI. A paucity of reports concerning different types of agent makes it difficult to establish which is truly effective. In a Cochrane review, Maeda and colleagues^14^ found only four eligible randomized trials^7^,^15^–^17^, including a total of 176 patients treated with injectable bulking agents: hydrogel cross-linked with polyacrylamide (Bulkamid™; Contura, Soeborg, Denmark)^7^, porcine dermal collagen (Permacol™; Covidien, Dublin, Ireland)^7^, polydimethylsiloxane elastomer implants^15^,^16^, silicone biomaterial (PTQ™; Uroplasty, Geleen, The Netherlands)^17^ and carbon-coated beads (Durasphere®; Carbon Medical Technologies, St Paul, Minnesota, USA)^17^. Unfortunately, the review authors found significant concerns of bias in all trials but one. They were unable to demonstrate significant effectiveness of perianal injection of bulking agents owing to the limited number of identified trials together with methodological weaknesses. Moreover, with limited follow-up (maximum 12 months) only a short-term benefit from injections was reported, regardless of the material used. A silicone biomaterial (PTQ™) provided some advantages and was safer in treating FI than carbon-coated beads (Durasphere®) in the short term. However, PTQ™ did not show obvious clinical benefit compared with normal saline injection. Delivery of the bulking agent under ultrasound guidance, compared with digital guidance, was more effective.

The major problem with other anal bulking agents used so far is their reduced efficacy with time, probably due to a variable combination of degradation and/or diffusion through the tissue adjacent to the injection site or, sometimes, far from that site (Contigen®, Bard, Covington, Georgia, USA; Zuidex™, Q-Med, Uppsala, Sweden). The present data suggest that the Gatekeeper™ overcomes all of these potential problems. In this preliminary experience, implantation of the Gatekeeper™ in the anal canal was safe, without any morbidity, either during or after surgery. The entire procedure was painless and well tolerated. Wounds healed in all patients and no discomfort was noted either at rest or on defaecation. Adverse events, sometimes severe, have been described for some other bulking agents used in urinary incontinence or FI, including pulmonary embolism, fatal stroke, periurethral mass and suspected disease transmission^18^–^21^. No adverse events were reported in the present series.

Of utmost importance was the significant improvement in episodes of major FI and both CCFIS and Vaizey score. Ability to postpone defaecation for longer and postevacuation soiling were both significantly improved in the majority of patients. Of note was the improvement in both SF-36® and FIQL scores in this series, suggesting regained health and quality of life in patients treated with Gatekeeper™. Furthermore, findings observed in the short and medium term appeared substantially unchanged at the final evaluation. This is important as some of the other bulking agents demonstrated very poor long-term results and have had no further clinical use despite preliminary reports suggesting good clinical effectiveness (polytetrafluoroethylene, autologous fat^1^,^2^).

This preliminary study demonstrated that the Gatekeeper™ anal implant was a safe, reliable and effective treatment for FI, with results maintained over time. These results call for larger series and longer follow-up better to establish the role of the Gatekeeper™ in the management of FI.
